# Benzene and other hazardous air pollutants in consumer-grade natural gas in Europe

**DOI:** 10.1088/1748-9326/ae499f

**Published:** 2026-03-25

**Authors:** Tamara L Sparks, Yannai S Kashtan, Sebastian T Rowland, Eric D Lebel, Jackson S W Goldman, Colin Finnegan, Gan Huang, Nicole Lucha, Abenezer Shankute, Nick Heath, Sofia Bisogno, Kelsey R Bilsback, Anchal Garg, Lee Ann L Hill, Robert B Jackson, Seth B C Shonkoff, Drew R Michanowicz

**Affiliations:** 1 PSE Healthy Energy, Oakland, CA, United States of America; 2 Department of Earth System Science, Stanford University, Stanford, CA, United States of America; 3 Woods Institute for the Environment and Precourt Institute for Energy, Stanford University, Stanford, CA, United States of America; 4 Environmental Health Sciences Division, School of Public Health, University of California, Berkeley, Berkeley, CA, United States of America; 5 Energy Technologies Area, Lawrence Berkeley National Lab, Berkeley, CA, United States of America

**Keywords:** benzene, natural gas, gas stove, gas leaks, hazardous air pollutants, indoor air quality, sulfur-based odorants

## Abstract

Consumer-grade natural gas leaks contribute to methane-induced climate change and can degrade air quality. However, limited leakage and gas composition data exist outside of North America. Here, we measured stove-off gas leakage in 35 homes and chemically characterized 78 unburned gas samples from residential stoves across seven cities in the United Kingdom, Netherlands, and Italy. On average, benzene in unburned gas was substantially elevated compared to North America (9 to 73 times higher), while sulfur-based odorants were lower. Modeling of indoor and outdoor benzene enhancements from gas leaks showed potential for hazardous benzene exposure, often undetectable by odor. Three of 35 homes exhibited a stove-off leak that, combined with city-median benzene in gas, resulted in modeled benzene enhancements above the European Union’s annual limit value (1.6 ppbv). The combination of high benzene and relatively low odorization in natural gas suggests that hazardous leaks are likely underreported in Europe.

## 1. Introduction

Natural gas leaks contribute to methane-induced climate change [1–5] and can be an underestimated source of air pollution and exposure in the event of behind-the-meter leakage [6–8]. Recent studies in North America have shown that indoor natural gas leaks contain numerous non-methane volatile organic compounds (NMVOCs), some of which are hazardous to human health [6–8]. The most toxic of these is benzene, exposure to which is associated with multiple adverse health outcomes, including cancer (specifically leukemia), impacts to the immune system (increasing risk of infection), and impacts to bone marrow and red blood cells that can lead to anemia and excessive bleeding [9–11]. Other hazardous components of natural gas, such as toluene, ethylbenzene, xylenes, and hexane, have additional neurological, developmental, and motor-function health impacts [12, 13]. Based on the widespread reliance on natural gas and the prevalence of leaks from household gas appliances [8, 14–17] and the extensive gas distribution pipeline network [18, 19], further chemical characterization of consumer-grade natural gas is needed, particularly outside North America, to estimate air quality impacts and risks to human health.

Concerns have been raised previously regarding benzene levels in pipeline gas sourced from the Netherlands and North Sea gas fields and the potential health impacts when this gas leaks [20, 21]. The National Institute for Public Health and the Environment (RIVM) in the Netherlands reported benzene levels in consumer-grade gas up to 0.1% w/w (238 ppmv) [21]. They provided a theoretical estimate of potential indoor air benzene enhancement from small leaks of gas from these fields of 280 *µ*g m^−3^ (90 ppbv), over 50 times higher than the European Union (EU) annual benzene limit value of 5 *µ*g m^−3^ (1.6 ppbv) [22] and over 150 times the World Health Organization (WHO) guideline of 1.7 *µ*g m^−3^ (0.53 ppbv) that represents a 1/100 000 excess lifetime risk of leukemia [9]. However, they did not measure actual leak rates or model exposure, estimating only based on how large a leak could be present without being detected by odor. They dismissed the risk from this scenario as unlikely but acknowledged that more information is required to adequately determine the health risk from small gas leaks. Marcogaz measured pipeline concentrations of benzene in gas up to 800 ppmv in gas from the Netherlands and the North Sea [20]. Using these data, Jacobs and Cornelissen [23] provided estimates of the potential indoor air benzene enhancement associated with stove-off gas leakage of 2.6 *µ*g m^−3^ (0.8 ppbv), which is higher than the WHO guideline [9] but below the EU limit [22]. However, this estimate relied on a single estimate of pipeline benzene reported over a decade ago, an average methane leakage rate derived from only six gas hobs measured in a laboratory setting, and a single air exchange rate. Notably, these pipeline benzene concentrations are substantially higher than any level observed throughout consumer-grade natural gas in the United States (US) and Canada [8].

To formally assess health risks from gas leaks, in addition to gas composition, leakage rates are requisite to establish an exposure pathway. Few data exist on behind-the-meter methane leakage outside the US. A recent US study estimated that gas stoves emit 28.1 Gg methane per year, with 76% of that originating from stoves sitting idle, with other US studies finding significant leakage in homes from stoves and other gas appliances [1, 5, 14]. While many studies have examined the importance of methane emissions from the oil and gas supply chain from a climate mitigation perspective [24–27], few studies have examined the direct air quality and potential health risks from indoor gas leaks. If portions of European consumer-grade natural gas in fact contain elevated benzene levels up to 800 ppmv [19, 20], routine gas leaks may be a larger source of benzene than previously understood.

When natural gas leaks, the only protective measure is odorants that are added to otherwise odorless gas. Odorization requirements vary widely but are typically designed so that a leak is detectable by a person with a normal sense of smell at 1% methane, a fifth of the lower explosive limit of 5% methane [28]. Previous work has found large variability in odorant concentrations in gas and that leaks that are too small to be detected by smell can result in hazardous benzene levels [8]. Given the sole reliance on odorants for detecting residential natural gas leaks, more data is also needed on odorant concentrations in consumer-grade natural gas to better determine what size leaks may escape detection via odor.

In this study, we report gas mole fractions (referred herein as ‘concentrations’ or ‘levels’) of natural gas chemical constituents, including benzene and sulfur-based odorants, in 78 natural gas samples from 35 residences across seven cities in the United Kingdom (UK), the Netherlands, and Italy. We report theoretical benzene enhancements as a function of detectable odor. We also report stove-off methane leakage rates for 35 stoves across the UK, Netherlands and Italy. We combine our observed leakage rates and benzene levels to predict indoor benzene enhancements using CONTAM, an indoor air quality model [29]. Finally, we model the likely outdoor benzene enhancement from a satellite-sensed super-emitting leak on a gas distribution pipeline in the UK [18].

## 2. Methods

### 2.1. Sample collection

We collected 78 samples of unburned natural gas from stoves in four cities in the UK (London, *n* = 12; St. Neots, *n* = 7; Manchester, *n* = 10; Edinburgh, *n* = 11), two cities in the Netherlands (Amsterdam *n* = 16, Leeuwarden *n* = 4), and one city in Italy (Milan, *n* = 18) between late 2023 and early 2024 (table S1). For data quality assurance, see the supplementary text. The countries we sampled in have higher gas stove usage compared to others in Europe, with 64% of energy used for cooking coming from natural gas in the Netherlands and 69% in Italy compared to 33% in the EU as a whole [30],and 54% in the UK [31].

We used sampling methodologies similar to previous campaigns [6–8]. We sampled gas directly from the stove’s gas outlet into lab-provided 1.0–1.4 l Silonite-lined canisters using PTFE tubing. We contracted a commercial analysis lab, ISO 17025 accredited Tera Environnement, who analyzed the natural gas content using gas chromatography mass spectroscopy for select NMVOCs, including 12 organosulfuric compounds that may be used as odorants. All samples were also analyzed for nitrogen, oxygen, methane, and ethane to verify sample capture. Samples were analyzed within a 40 d hold time to minimize degradation of samples. We assigned values below the detection limit a value of zero, except in the specific case with tert-butyl mercaptan (TBM) discussed in the Results section.

### 2.2. Stove methane emission measurements

We measured 35 individual stove methane leakage rates in the UK (*n* = 12), the Netherlands (*n* = 13), and Italy (*n* = 10) using the protocol of Lebel *et al* [5] except for using N_2_O as the tracer to estimate air exchange. Briefly, we enclosed each kitchen with plastic sheeting to create a chamber of discoverable volume to facilitate leakage rate measurements. Two fans were placed in the kitchen to ensure mixing. We then injected a known volume of N_2_O into the kitchen as a tracer gas to calculate the air exchange rate and subsequent chamber volume. With the gas stove turned off, we continuously measured methane concentrations in the chamber for at least 5 min to quantify the rate of concentration increase. Methane and N_2_O were measured using an Aeris Technologies MIRA Ultra calibrated with gas standards. Using the known kitchen volume and air exchange rate, we then converted this rate into an estimate of methane leakage. We correct methane estimates for the air change rate because if there is a high air change between the chamber and outside the chamber, the measured leak will be underestimated. For each kitchen chamber, we conducted a minimum of three tracer gas tests and three leak tests and averaged the results. In a handful of cases the uncertainty produced negative leak rate values. Such values are expected when the actual leak rate was small and the baseline methane concentration was slightly elevated due to atmospheric conditions. Although a negative leak rate is not physically possible, we retained these values in our analysis under the assumption that measurement error was symmetrically distributed (see supplementary text).

### 2.3. Modeling indoor air quality with CONTAM

We used CONTAM, a multizone indoor air quality model developed by the National Institute of Standards and Technology (NIST), to model the indoor benzene and odorant enhancements associated with natural gas leaks from stoves. We assume that the leak is governed by bulk flow, so the benzene-to-methane ratio in leaked gas is the same as what we measured in the pipe (see supplementary text for sensitivity analysis). CONTAM has recently been used to model indoor air quality in European locations, including Trondheim and Wallonia [32],and has been validated for modeling indoor concentrations of benzene [33] and NO_2_ [34] from stove emissions in the US. We selected the APT-62 model floor plan from among those constructed in CONTAM by Persily *et al* [35],whose living area of 65 m^2^ was within 40% of the median living area in each study city **[**36**–**38**]**.

The model floor plan was left unchanged except for the modifications described by Kashtan *et al* [34]. Briefly, we modified Persily *et al*’s floor plans by adding one National Fenestration Research Council standard window (1.2 m by 1.5 m or 4 ft. by 5 ft.) [39] to an exterior wall in every bedroom, living room, and kitchen and replacing interior doors with fixed bi-directional airflow to simulate open doors. The central forced-air system was left unchanged. In addition to these changes, we used median literature parameter values for airtightness and ventilation reported for each study city (see table S2 for a summary of the parameters used).

For each city’s representative floor plan and parameter set, we modeled kitchen benzene concentrations for the median, 75th percentile, and 95th percentile stove leakage rates using the city’s median benzene-to-methane ratio that we measured in homes there. We modeled the resulting stove-off kitchen concentrations under different air exchange regimes, including having all windows closed, the kitchen window open 4 or 24 h a day, and with all windows closed but with a central air conditioner running. Because we observed a linear function relating stove leakage emission rates to resulting kitchen benzene enhancements, we utilized this linear function to produce benzene enhancements for individual homes and their respective measured empirical leak rates.

We also modeled kitchen benzene enhancements corresponding to various detectable odorant enhancements. We followed a similar protocol for determining odorant detection thresholds as in Rowland *et al* [8]. Briefly, among the odorants measured in a given country’s gas, we first determined the odorant with the highest concentration-to-detection-threshold ratio. We assumed that this was the primary odorant (see supplementary text for further details). We then modeled the kitchen benzene concentration corresponding to a threshold where 50% of people could detect an odor (DT_50_), as reported by Nagata for the primary odorant at the median odorization in a given country [40].

We grouped methane leak rates across Europe because leak rates from different cities and countries within Europe were statistically indistinguishable according to a two-tailed Mann-Whitney U test (p between 0.37 and 0.89). However, benzene concentrations in natural gas were statistically different across many city pairs in Europe (with many *p* values ≪ 0.01), and odorization levels were statistically different across countries in Europe (*p* ≪ 0.01); therefore, we grouped these parameters by city or country accordingly. See supplementary text for uncertainty analysis.

### 2.4. Outdoor benzene enhancement from a pipeline leak using American Meteorological Society/Environmental Protection Agency Regulatory Model (AERMOD)

To assess the potential health impacts of outdoor leak events, we modeled a leak from a low-pressure gas distribution pipeline near Cheltenham, UK, in spring 2023 [18]. We simulated the leak using a combination of satellite-derived emission estimates, simulated meteorology, and dispersion modeling. Satellite estimates of leak emission rates were obtained from the GHGSat constellation. GHGSat first detected the leak on 27 March 2023, and observed it four more times through June 12. Using these observations, Dowd *et al* used the integrated mass enhancement method to estimate emission rates ranging from 236 to 1,375 kg h^−1^over a three-month period [18]. The following satellite-derived estimates were used as model inputs:

27 March—19 April: 236 kg h^−1^
20 April—19 May: 1071 kg h^−1^
20 May—21 May: 1375 kg h^−1^
22 May—6 June: 438 kg h^−1^
7 June—13 June: 290 kg h^−1^

The AERMOD was used to model source-attributable hourly ground-level outdoor air concentrations [41, 42]. The leak was modeled as a 10-by-10 m volume source based on the location uncertainty presented by Dowd *et al* [18]. AERMOD receptors were placed with a 5 m spacing on a pseudo fence line around the leak, at a 25 m spacing between the fence line and 300 m from the source, at a 100 m spacing from 300 m to 1 km, 500 m spacing from 1 km to 5 km, and 1000 m spacing from 5 km to 50 km, based on regulatory guidance. This configuration resulted in approximately 9100 receptors. Transport and dispersion were driven by hourly meteorological data from the Weather Research and Forecasting (WRF) model. The initial and boundary conditions for the WRF were obtained from ECMWF Reanalysis v5 (ERA5). Our WRF simulations used a 4 km grid spacing with 35 vertical layers and employed four-dimensional data assimilation (FDDA) using ERA5 analyses. The FDDA nudges the temperature, winds, and humidity above the boundary layer toward the ERA5 analyses to help constrain the model and to maintain consistency with observations over the simulated period [43].

Using the methane emission rates and resulting concentrations from AERMOD, we applied a median benzene-to-methane molar ratio of 1.15 × 10^−4^ (mol mol^−1^) to simulate source-attributable benzene concentrations. The leak originated from a low-pressure distribution pipeline, suggesting that the consumer-grade natural gas composition data collected in the UK were likely representative of what was released during this leak. This ratio was determined as the median benzene-to-methane ratio of samples from London and St. Neots. Given the north-south trend in benzene in the UK, we chose these samples as they are closest in latitude to the leak and our best estimate of the leak’s gas composition. Given their atmospheric lifetimes, chemical degradation of benzene and methane is negligible on the 1 h modeled timescale.

The benzene-to-methane molar ratio (mol mol^−1^) at the source was preserved at all receptor locations because AERMOD simulates dispersion independent of the molecular properties of the dispersed pollutant. Thus, we estimated the one-hour average concentrations of benzene in ppbv (*C*_benzene_) using the following formula:

\begin{align*}{C_{{\mathrm{benzene}}}} &amp;= {C_{{\mathrm{methane}}}} \cdot {R_{{\mathrm{cons}}}} \cdot {T_{{\mathrm{STP}}}} \cdot {R_{{\mathrm{benzene:methane}}}} \nonumber\\ &amp;\quad \cdot{P_{{\mathrm{STP}}}}^{ - 1} \cdot {{M}}{{{W}}_{{\mathrm{methane}}}}^{ - 1} \cdot 1000\end{align*}

where *C*_methane_ is the AERMOD-modeled methane concentration (*μ*g m^−3^), *R*_benzene:methane_ is the molar fraction of benzene to methane, *R*_cons_ is the ideal gas law constant (8.2 × 10^−5^ m^3^ atm K^−1^mol^−1^), *T*_STP_ is the temperature (K) at standard temperature and pressure (STP), *P*_STP_ is pressure (atm) at STP, and *MW*_methane_ is the molecular weight of methane (g mol^−1^).

No direct benzene measurements were available to validate the model, but AERMOD has been extensively validated and has been similarly used to model benzene health impacts from oil and gas activity [43].

## 3. Results and discussion

### 3.1. Hazardous air pollutants in consumer-grade natural gas

**Figure 1.**
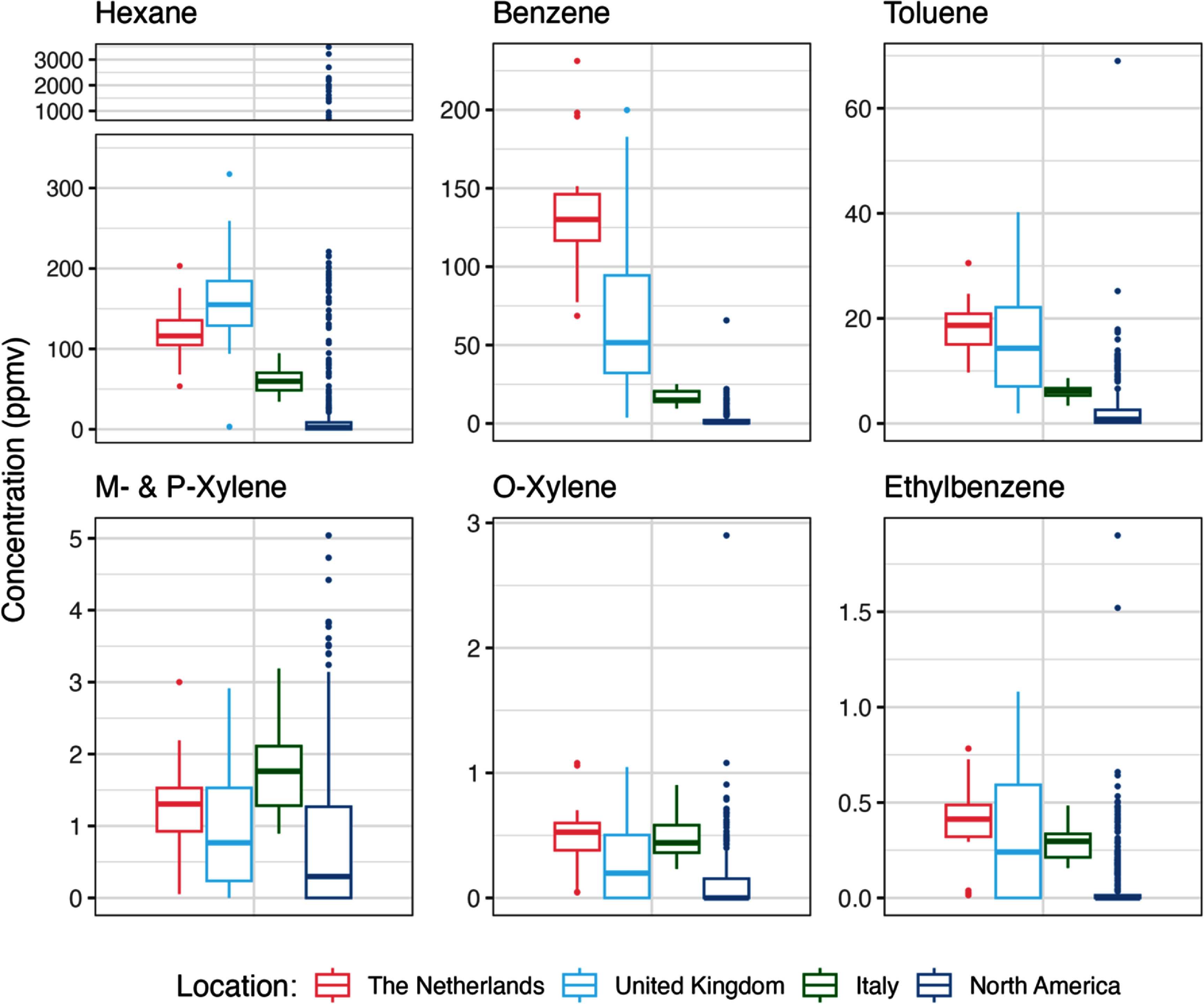
Box-and-whisker plot of hazardous air pollutants measured in unburned natural gas in the Netherlands, the United Kingdom, and Italy (from this study) and North America (United States and Canada) from Rowland et al. [8]. The plot for hexane has a broken axis to display the unusually high concentrations found in Washington, DC. Differences between each location for each compound were statistically significant using a pairwise Mann-Whitney U test with Bonferroni correction for all combinations (*p* values < 0.02) except between the Netherlands and the UK for toluene, *m*- and *p*-xylene, and ethylbenzene (*p* values > 0.70), and o-xylene (*p* = 0.06), between the Netherlands and Italy for *m*- and *p*-xylene, *o*-xylene, and ethylbenzene (*p* values >0.33), between the UK and Italy for ethylbenzene (*p* = 1.00), and between the UK and North America for m- and *p*-xylene (*p* = 0.05).

Hexane, benzene, and toluene were detected in 100% of samples. Ethylbenzene, *m*- and *p*-xylene, and *o*-xylene were detected in 100% of samples in the Netherlands and Italy, and in most samples in the UK (68% for ethylbenzene, 92% for *m*- and *p*-xylene, and 70% for *o*-xylene). The average concentrations of these hazardous air pollutants in all three countries were substantially elevated compared to gas samples previously collected in North America, particularly for benzene (figure 1; table S3). For benzene, the average concentrations were 66, 37, and 8.6 times higher than North America for the Netherlands, UK, and Italy, respectively (comparing against measurements in the US and Canada from Rowland *et al* [8],which also incorporates measurements from Lebel *et al* [7] and Michanowicz *et al* [6]). The other hazardous air pollutants ranged from 2.3–8.1 times higher in the Netherlands, 1.7–7.6 times higher in the UK, and 2.7–6.2 times higher in Italy. This indicates that gas delivered to customers in these regions is systematically higher in terms of hazardous air pollutant makeup compared to North America.

**Figure 2.**
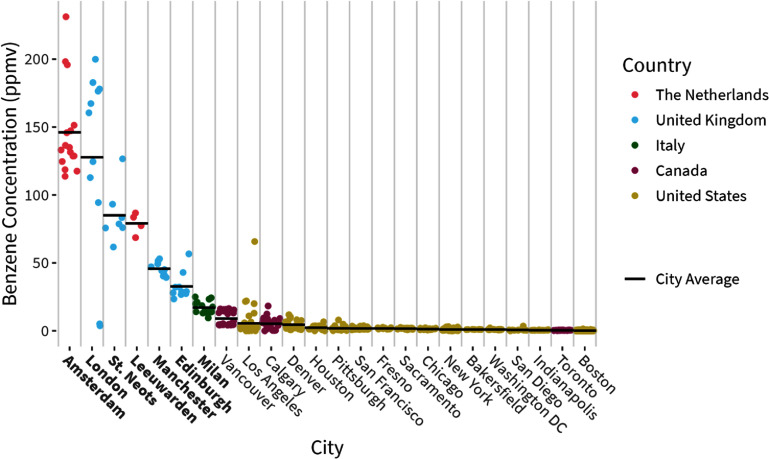
Benzene concentrations measured in unburned, consumer-grade natural gas samples in cities in Europe (this study, in bold) and North America (data from Rowland et al. [8]), ordered by decreasing average concentration by city.

We found that benzene concentrations in natural gas in Europe were the highest relative to values in North America and the most distinct between European cities (figure 2; table S4). Of the cities sampled, Amsterdam had the highest average benzene concentration measured, with an average of 146 [95% CI: 132, 163] ppmv, which is 73 times higher than the North American average of 2 ppmv and at least 16 times higher than any single city average in North America [8]. In the Netherlands, Leeuwarden had an average benzene concentration of 79 [95% CI: 72, 85] ppmv, almost 40 times higher than that in North America. Similarly, elevated benzene concentrations were observed across cities in the UK, ranging from 33 to 128 ppmv, 17–64 times higher than the North American average, with a geographic trend whereby benzene concentrations nearly quadrupled moving from north (Edinburgh) to south (London) (see figure 2). In Milan, average benzene levels were 17 [95% CI: 15, 19] ppmv, which is lower than that of other European cities, but still nearly nine times higher than the average concentration in North America. It is likely that these higher levels across Europe are attributable to higher benzene concentrations that have been observed in gas sourced from the Netherlands and North Sea gas fields [20].

### 3.2. Theoretical benzene exposure as a function of gas leak odor

**Figure 3.**
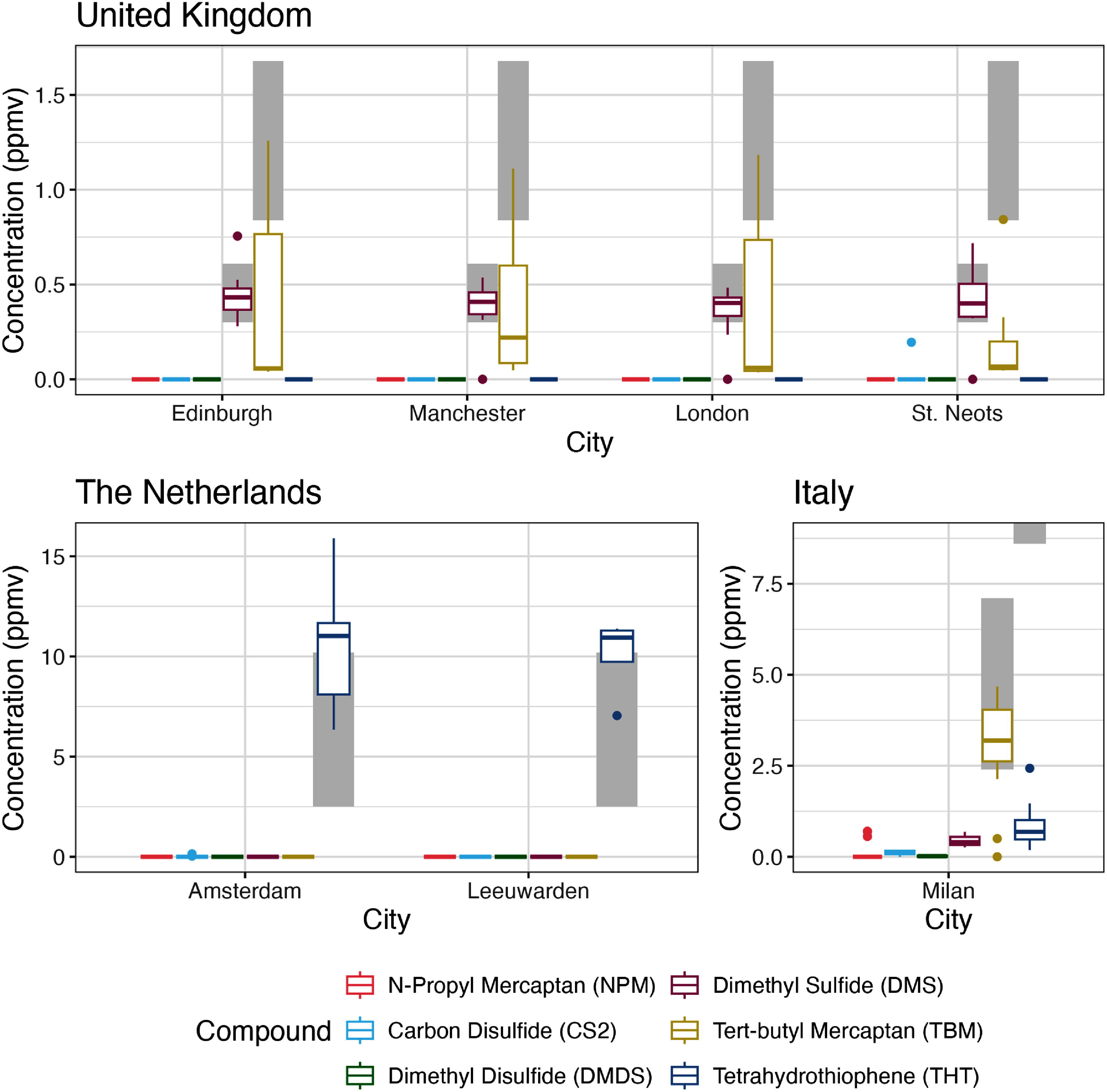
Box-and-whisker plot of odorant concentrations by city for the United Kingdom, the Netherlands, and Italy, including only odorants that were detected in any city. Gray boxes indicate each country’s guidelines for the range of odorant concentrations in their natural gas systems.

We detected sulfur-based odorants in all but two natural gas samples collected with distinct profiles for each country (figure 3). On average, Italy’s odorization was on par with North American cities, while odorization in the Netherlands and the UK was lower (see supplementary text) [8]. When comparing to natural gas odorization practices and guidelines for Europe, an average tetrahydrothiophene (THT) level of 10.3 [95% CI: 9.2, 11.3] ppmv in the Netherlands fell just above their recommended range of 2.5–10.2 ppmv [28, 44]. Odorants measured in Italy generally met odorization guidelines for tert-butyl mercaptan (TBM) concentrations (3.1 [95% CI: 2.3, 3.7] ppmv compared to a recommended range of 2.4–7.1 ppmv), but did indicate attenuated average THT levels (0.8 [95% CI: 0.6, 1.1] ppmv vs a recommended minimum concentration of 8.6 ppmv [28]. Of the 37 UK samples, 34 (92%) contained dimethyl sulfide (DMS), 16 (43%) contained TBM, one (3%) contained carbon disulfide (CS2), and two (5%) did not have any detected odorants. The UK’s gas policy calls for odorization with a blend of 80% TBM and 20% DMS at 6 ± 2 mg m^−3^ [28, 45]. Because TBM and DMS are assumed to be added as a pre-mixture and the lowest detected TBM values were near the detection limit, we presumed that TBM was present but below detection in the undetected samples. Because we could not quantify the TBM in these samples, we set their value equal to the detection limit divided by two [46] (see supplementary text for sensitivity analysis). Based on this assumption, the average sum of TBM and DMS was 2.3 [95% CI: 1.8, 2.9] mg m^−3^, a level approximately three times lower than the ‘typical concentration’ reported in UK’s gas odorization policy, due primarily to loss of TBM (figure 3) [47].

To extrapolate theoretical benzene concentrations that could persist just below its accompanying human-based odor threshold, we first determined relative odor strength as the ratio of odorant concentration to the DT_50_ detection threshold for each city (see supplementary text and table S5) [40]. Italy recorded the strongest relative odorization levels, followed by the Netherlands and UK which recorded relative odorization at nearly an order of magnitude lower than Italy. Due to the lower odorization levels observed in the UK, benzene concentrations from a gas leak could reach an elevated 62 ppbv in London (along with 421 ppmv methane) before accompanying odor levels would cross the detection threshold—almost 40 times the annual EU benzene limit value of 1.6 ppbv [22] (but well below the 50 000 ppmv methane explosivity threshold [28]). Similarly elevated benzene levels could persist in other UK cities with 39, 15, and 7.0 ppbv benzene predicted in St. Neots, Edinburgh, and Manchester, respectively. In the Netherlands, indoor benzene levels could reach 8.3 ppbv in Amsterdam and 4.7 ppbv in Leeuwarden before the primary odorant, THT, crossed its odorant detection threshold. These theoretical benzene enhancements are lower than the 90 ppbv posed by RIVM for the Netherlands using a similar DT_50_ (1 ppbv vs 0.62 here), as our median measured benzene in gas was much lower than their estimate that explicitly used high-end rather than average benzene levels [21]. Conversely, Milan with its relatively higher odor content results in a theoretical odorless benzene level of just 0.16 ppbv—well below the EU limit value. Overall, the combination of high benzene and low odorization suggests that relatively large and unsuspecting gas leaks could persist, accompanied by serious benzene exposures (table S6).

### 3.3. Stove-off methane leakage rates

**Figure 4.**
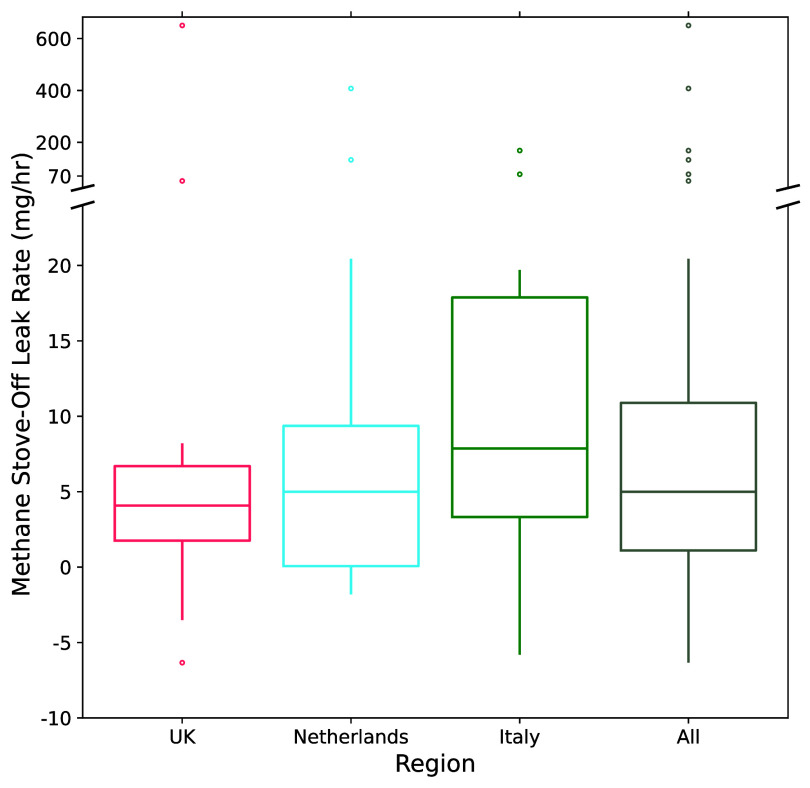
Box-and-whisker plot of methane stove-off leak rates, represented with a broken axis due to the long-tailed distribution. Duplicate measurements of the same stove are averaged together. Negative values are present due to measurement error (see methods).

We measured methane leakage from 35 residential stoves while turned off across Europe (figure 4; table S7). We found a long-tailed distribution with mean and median methane stove-off leak rates of 46 [95% CI: 18, 120] and 5.0 [95% CI: 2.5, 8.2] mg h^−1^, respectively, and a range from no detectable leak to 651 mg h^−1^. Leak rates between countries were not statistically distinguishable from each other (*p* = 0.16 or greater for all pairwise Mann-Whitney U tests). These results agree with the previously reported stove-off methane leak rates from Jacobs and Cornelissen who found a mean and median of 56 [95% CI: 22, 92] and 60 [95% CI: 4,104] mg h^−1^, respectively, from six gas stoves in a lab setting in the Netherlands [23]. Their leak rates were also highly variable, even in a controlled lab setting, ranging from no detectable leak to 130 mg h^−1^. Together these findings suggest that there is wide variability in stove leak rates, with a portion of stoves leaking two orders of magnitude greater than others.

Previously measured stove-off methane leakage from residential kitchens in California had a mean and median of 58 [95% CI: 39, 90] mg h^−1^ and 24 [95% CI: 16, 30] mg h^−1^, with a similarly long-tailed distribution [5]. Our results suggest that the median leak rate of European stoves may be significantly lower than that of their California counterparts (*p* < 0.001 for a pairwise Mann-Whitney *U* test); however, further work is required to confirm this pattern due to the small sample size (table S8). If real, this difference may be due to regulations in Europe that require a certified professional to install gas appliances [48].

### 3.4. CONTAM modeled indoor benzene enhancement

**Figure 5.**
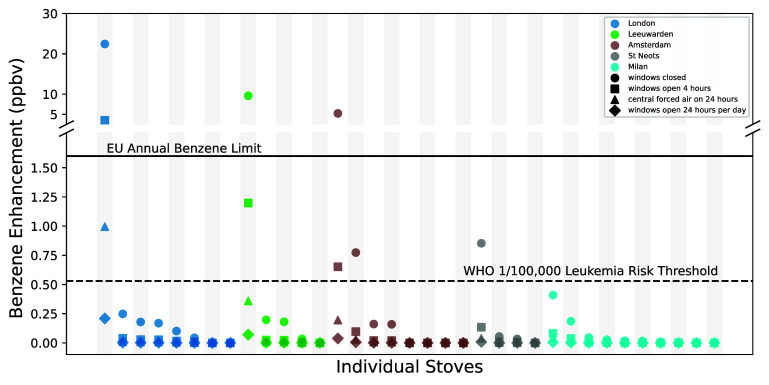
Modeled kitchen benzene enhancements associated with measured stove-off leak rates and city-median benzene levels in cities where leak rates were measured. Colors indicate the city modeled while shapes indicate the ventilation conditions (see legend). Each column (series of four points) represents the gas leak rate measured from a single stove. The benzene-to-methane ratio used is the median of the city in question and the floor plan parameters are selected based on the country (see methods). Modeled negative values (> −0.16 ppbv) due to negative leak rates (see supplementary text) are clipped to zero in the plot but are included in all summary statistics. The solid line represents the European Union (EU) ambient annual benzene exposure limit value (1.6 ppbv) [22]. The dotted line represents the concentration of benzene exposure corresponding to the World Health Organization (WHO) 1/100 000 increased lifetime leukemia risk (0.53 ppbv) [9].

Across all the stoves tested and modeled, the median elevated indoor benzene concentration was 0.04 [95% CI: 0.006, 0.17] ppbv, well below the EU and WHO health benchmarks (see supplementary text for details on health benchmarks) [9, 22]. However, we identified three of the 35 (∼9%) European stoves tested with stove-off gas leakage rates of particular concern. In a typical ∼65 m^2^ apartment with city-median natural gas-benzene concentrations, leakage rates observed for these three stoves would be sufficient to raise indoor benzene levels several times above the EU annual limit value of 1.6 ppbv [22] (figure 5). While modeled benzene concentrations were highly sensitive to air exchange rates (figure 5; table S9), the highest modeled indoor benzene enhancement was for a stove in London, where we estimated a 22 [95% CI: 14, 45] ppbv enhancement—over 13 times the annual EU limit value. Even in a scenario with moderate air exchange (i.e. windows open for four hours per day), we observe an enhancement of 3.5 [95% CI: 2.2, 7.1] ppbv. Notably, the modeled odorant enhancement from this same stove fell below the DT_50_ detection threshold for all air exchange scenarios indicating that this leak would more likely than not go unnoticed. Indeed, no odor was reported by researchers during sampling at this location. It is important to note these modeled benzene exposures are conservative as they do not include background sources of benzene, such as benzene produced from natural gas combustion [49], outdoor gasoline combustion [50], or tobacco smoke. This exposure pathway may explain at least a portion of an exposure discrepancy observed by Kotzias, who found differences between the daily benzene inhalation exposure and the sum of known exposures measured in individual indoor environments across eleven European cities [51].

Compared to RIVM’s [21] and our own theoretical benzene exposure estimates based just on odorization levels, here we have used actual stove leak rates and indoor air modeling to better constrain the potential health risks associated with benzene exposure from gas stove leaks. We found measured stove leaks that, with limited air exchange, could result in benzene levels that exceed the EU limit but are below the threshold of odor detection, a scenario the RIVM report claimed was unlikely but had no evidence for or against [21]. While the Groningen gas field in the Netherlands has ceased production [52],using the reported benzene levels in Groningen gas of 0.075% w/w (179 ppmv) with our model of the leakiest stove in the Netherlands (410 mg h^−1^ methane) would result in a 22 ppbv enhancement of benzene, over 13 times the 1.6 ppbv EU limit. This suggests that the health risk from benzene exposure was previously higher if those levels of benzene were indeed delivered to consumers as reported [21].

### 3.5. AERMOD modeled outdoor benzene enhancement from a pipeline leak

Given the elevated benzene levels in consumer-grade natural gas, we modeled outdoor benzene enhancement associated with a super-emitting gas leak near a residential area in the UK [18]. We found good agreement of plume characteristics throughout the 11 week leak period between the AERMOD air quality dispersion model and available GHGSat observations (figure S1) [53]. Given the protracted nature of the leak, we compared modeled ground level benzene enhancements to both the EU ambient annual limit value of 1.6 ppbv and the 2026 EU 8 h occupational exposure limit value of 200 ppbv [54].

**Figure 6.**
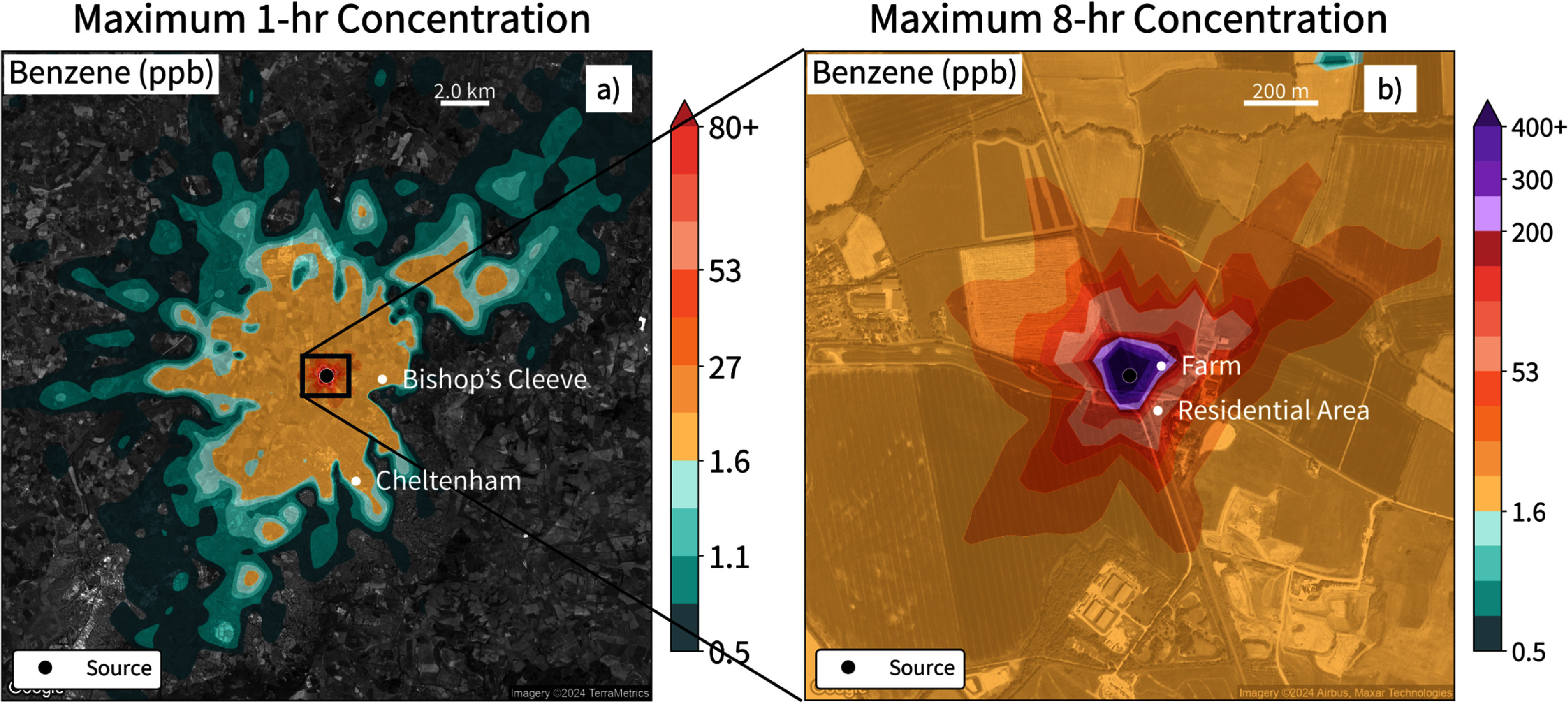
Modeled source-attributable surface air benzene concentrations around the Cheltenham leak. Spatially interpolated values based on variable receptor spacing shown on the maps represent the maximum of the (a) 1 h or (b) 8 h averaged benzene concentrations during the modeled period. The widespread impact is shown on the left (a) and the zoomed-in panel on the right (b) shows the immediate impact near the leak. Shades of purple in (b) show where 8 h benzene concentrations exceed the European Union (EU) occupational exposure limit of 200 ppbv [54]. The location of the leak is shown as a black dot in both panels.

The maximum modeled 1 h benzene enhancement was 1277 ppbv ∼50 m downwind of the leak (figure 6(a)). In our simulations, benzene enhancements near the source exceeded 1.6 ppbv for 60% of the 11 weeks, and when averaged over an entire year with a zero-benzene background, exceeded the annual limit for the year. The maximum 8 h benzene concentration was 855 ppbv, more than four times the 200 ppbv occupational exposure limit, suggesting that exposure could have been substantial to workers at the leak site if appropriate personal protective equipment was not used. The occupational limit value was also reached for at least one 8 h period 140 m from the source, an area that includes a small farm ∼70 m away, and part of a small residential area within 200 m (figure 6(b)). Moreover, this leak likely affected a wide-ranging area downwind. The maximum modeled distance that a benzene enhancement of 1.6 ppbv was reached for at least 1 h was 10 km from the source—an area that includes several villages and the town of Cheltenham (figure 6(a)). The maximum modeled 1 h benzene enhancement at 2 km from the leak over one of the larger villages, Bishop’s Cleeve (population ∼14 000), was 5.8 ppbv. This indicates that super-emitter type leaks can cause general population exposures at considerable distances, while acute occupational exposures can be substantial for pipeline workers.

## 4. Conclusion

Because natural gas contains mostly methane, problems arising from gas leaks are often framed in terms of climate change or explosion risk [1–4, 55, 56]. Framing leaks solely in these terms, however, misses a key risk: leaks can contain benzene at sufficiently high concentrations to exceed health benchmarks in the nearby environment [7, 8, 23, 57]. We found that natural gas in Europe contains far higher levels of benzene, up to 73 times greater, than those measured in three recent studies in North America [6–8]. Modeled enhancements of benzene were sufficiently high to result in kitchen benzene concentrations exceeding the EU limit by several fold in cases with minimal air exchange, relatively high stove-off leak rates, and high city-median benzene concentrations. Substantial benzene enhancements are also not limited to indoor environments. Using satellite remote sensing, we showed that a large outdoor gas leak can produce benzene levels that can exceed both the EU annual and occupational exposure limits.

While odorant additives are intended to attenuate gas leak explosion risks [21, 28], they also could alert against smaller leaks and subsequent benzene exposures. Most of the benzene enhancements from leaks we modeled would likely be undetectable by smell, and the theoretical benzene enhancements that would evade odor detection in the UK and the Netherlands are well above health benchmarks—almost 40 times the annual EU benzene limit value based on data collected in London. Overall, gas data collected herein indicates that odorization levels are insufficient to alert against leaks containing elevated benzene, particularly in the UK. While increasing odorization would help warn against benzene exposure, and there is precedent for higher odorant concentrations [58], potential exposure risks from odorants themselves should be considered [59].

Additional research could enhance future estimates of benzene exposure associated with gas infrastructure in several ways: first, by incorporating leakage from other appliances like gas water heaters and furnaces; second, by incorporating benzene produced during gas combustion while operating stoves [34]; third, by expanding the geographical reach of this work; and fourth, by observing gas composition over longer periods of time, as composition can change seasonally and when different sources of gas are used.

Throughout Europe, successful policies have substantially reduced emissions of benzene and other air pollutants in the general environment [60]. The EU recently released a new directive on cleaner air for Europe which calls for a further reduction of the annual benzene limit value from 5 to 3.4 *μ*g m^−3^ (1.6 to 1.1 ppbv) by 2030 [61]. Our findings show that routine leaks within the natural gas supply chain can result in indoor and outdoor benzene levels surpassing existing and future EU air quality guidelines, posing a challenge to the EU’s goal of safeguarding human health.

## Data Availability

The data that support the findings of this study will be openly available at the following URL/DOI: https://zenodo.org/records/15643041 [62]. Supplementary Materials available at https://doi.org/10.1088/1748-9326/ae499f/data1.
